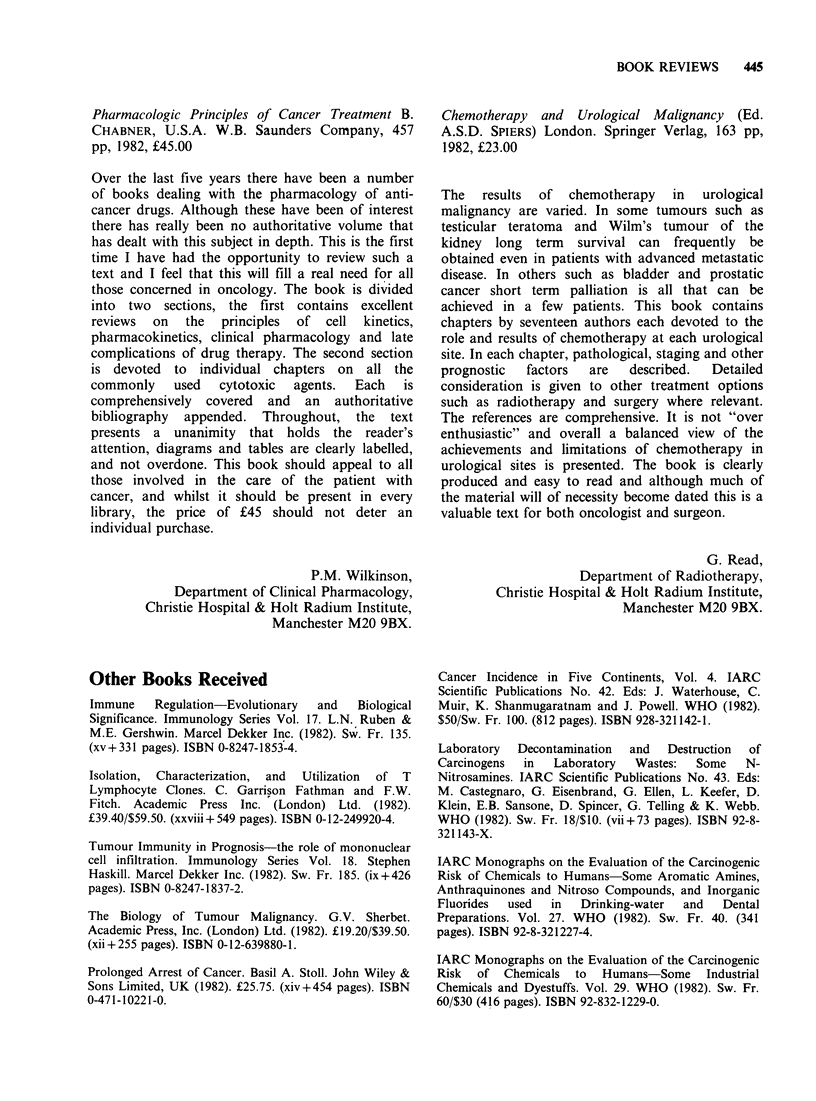# Chemotherapy and Urological Malignancy

**Published:** 1983-03

**Authors:** G. Read


Christie Hospital & Holt Radium Institute,

Manchester M20 9BX.

*Chemotherapy and Urological Malignancy* (Ed. A.S.D. SPIERS) London. Springer Verlag, 163 pp, 1982, £23.00

The results of chemotherapy in urological malignancy are varied. In some tumours such as testicular teratoma and Wilm's tumour of the kidney long term survival can frequently be obtained even in patients with advanced metastatic disease. In others such as bladder and prostatic cancer short term palliation is all that can be achieved in a few patients. This book contains chapters by seventeen authors each devoted to the role and results of chemotherapy at each urological site. In each chapter, pathological, staging and other prognostic factors are described. Detailed consideration is given to other treatment options such as radiotherapy and surgery where relevant. The references are comprehensive. It is not "over enthusiastic" and overall a balanced view of the achievements and limitations of chemotherapy in urological sites is presented. The book is clearly produced and easy to read and although much of the material will of necessity become dated this is a valuable text for both oncologist and surgeon.

G. Read,
Department of Radiotherapy,
Christie Hospital & Holt Radium Institute,
Manchester M20 9BX.